# A case of blastic plasmacytoid dendritic cell neoplasm in a young male with atypical cutaneous findings

**DOI:** 10.1016/j.jdcr.2025.10.014

**Published:** 2025-10-17

**Authors:** Olamide Sonuga, Aneri B. Patel, Briana Halle Brady, Linda Doan, Janellen Smith

**Affiliations:** a Department of Dermatology, University of California, Irvine, California; b Department of Dermatology, UC Davis School of Medicine, Sacramento, California

**Keywords:** blastic plasmacytoid dendritic cell neoplasm, BPDCN

## Introduction

Blastic plasmacytoid dendritic cell (pDC) neoplasm (BPDCN) is a rare myeloid cancer without a standard treatment. It comprises 0.44% of blood cancers annually, with an estimated 700 US cases.^1^ BPDCN arises from the malignant transformation of pDCs, immune cells involved in antiviral defense.^1^ Genetic mutations and epigenetic alterations disrupt normal pDC differentiation, driving aggressive progression and poor prognosis.^2^ BPDCN often presents with skin lesions—sometimes without other organ involvement—before progressing to a ‘leukemic phase,’ with infiltration of the bone marrow and bloodstream.^2^ The median age at diagnosis is 65 to 67 years.^3^ Because the clinical presentation may resemble benign skin conditions or other hematologic malignancies, accurate and timely diagnosis is critical to optimizing outcomes. We reported an unusual case of BPDCN in an 18-year-old male with skin findings on an individual with skin type III to IV, and reviewed the literature.

## Case report

**Fig 1:**
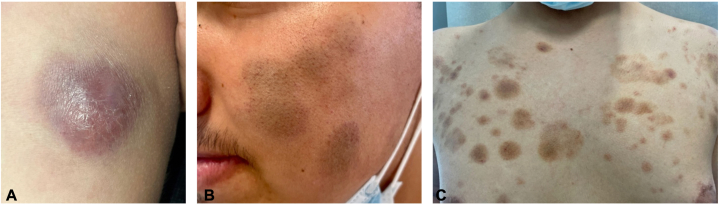
Clinical features of blastic plasmacytoid dendritic cell neoplasm. **A,** An 8 cm raised red-to-purple nodule with surrounding mild erythema present on the arm. **B,** Three ill-defined hyperpigmented patches 5 to 6 cm in size present on the left cheek. **C,** Numerous 2 to 3 cm hyperpigmented, brown macules are present on the chest.

An 18-year-old male with skin type III to IV presented with an approximately 4-cm bluish-purple mass on his right arm (Fig 1, *A*), accompanied by elbow swelling. Over 3 months, he developed multiple brown patches on his face, trunk, and extremities (Fig 1, *B* and *C*). He reported fatigue but denied other systemic symptoms of fever, weight loss, or lymphadenopathy.

**Fig 2:**
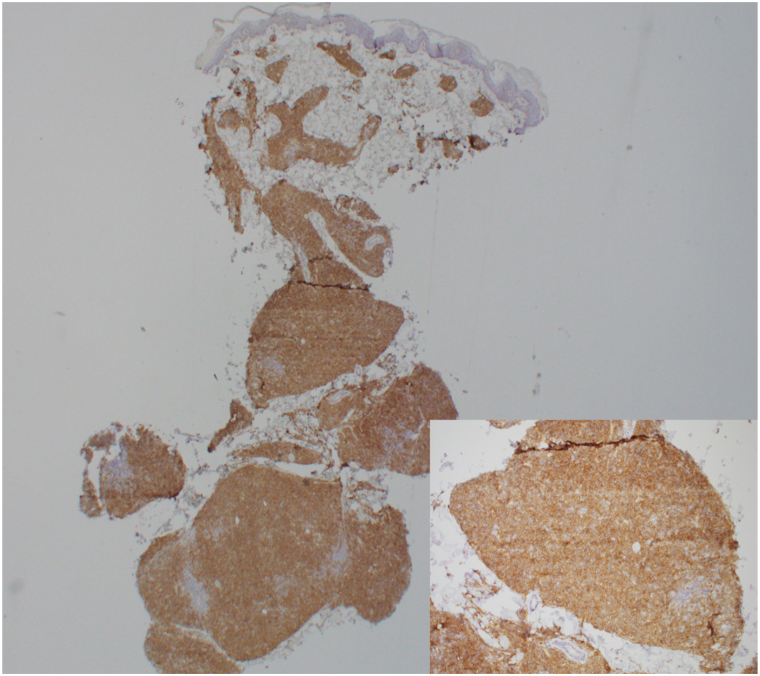
Histologic features of blastic plasmacytoid dendritic cell neoplasm (BPDCN) with CD123. (Original magnifications: ×2 and ×10.) *CD,* Clusters of differentiation.

A biopsy of a violaceous nodule on the left ventral proximal forearm revealed a dense dermal infiltration composed of intermediate-to-large cells with irregular nuclear contours and open, watery chromatin with scant cytoplasm. The atypical cells were positive for clusters of differentiation (CD) 4, CD56, and CD123, and negative for CD3 and myeloperoxidase. Epstein-Barr encoding region in situ studies were negative. The histologic findings overall were compatible with a BPDCN (Fig 2). Laboratory evaluation showed elevated levels of lactate dehydrogenase and white blood cell counts, with a neutrophilic and lymphocytic predominance. Lumbar puncture was negative for BPDCN. Positron emission tomography-computed tomography (PET-CT) scan revealed lymphadenopathy, splenomegaly, and multiple fluorodeoxyglucose-avid skin lesions consistent with BPDCN.

The patient was initially treated with systemic corticosteroids, followed by 2 cycles of hyperfractionated cyclophosphamide, vincristine, adriamycin, dexamethasone, methotrexate, and cytarabine plus venetoclax. A subsequent PET-CT scan and bone marrow biopsy 2 months after initiation of chemotherapy revealed significant disease improvement. The patient later received a successful stem cell transplant from his human leukocyte antigen-matched sibling.

As of this writing, he is 18 months after a stem cell transplant, and he continues to demonstrate positive clinical progress. He has returned to school and is working part-time. He remains asymptomatic, denying fever, chest pain, shortness of breath, cough, sore throat, nausea, or abdominal pain. White blood cell counts remain stable. A year after the stem cell transplant PET-CT scan showed nonspecific uptake in the tonsillar, cervical, and axillary lymph nodes, thought to be reactive after an upper respiratory infection. A mild fluorodeoxyglucose-avid skin lesion on the upper back was deemed likely inflammatory, as no corresponding new lesion was observed on physical examination.

## Discussion

BPDCN is a rare, aggressive malignancy with hallmark cutaneous signs. Literature typically describes skin lesions as purple-blue nodules, plaques, or patches, often on the head, face, and extremities. This case presents an atypical clinical picture, with coexisting hyperpigmented patches and a classic nodule in the absence of systemic symptoms. It adds value to the limited photographic representation of this condition in patients with darker skin phototypes. Early biopsy should be considered when encountering atypical morphologies to ensure rare variants are not overlooked.

Current treatment includes rapid, high-dose induction therapy modeled after acute myeloid leukemia regimens, with some patients proceeding to allogeneic stem cell transplant. Interdisciplinary care with hematology, oncology, and dermatology is essential. If the disease is refractory to acute myeloid leukemia-based regimens, switching to an aggressive alternative—preferably an acute lymphocytic leukemia regimen including asparaginase—is recommended, with agents like clofarabine or methotrexate as options.^4^^,^^5^ Local radiation may help with quality-of-life-impairing lesions or complement chemotherapy.

Targeted therapies like Tagraxofusp-erz (SL-401, an interleukin-3/CD123-directed agent) show promise,^2^^,^^6^ but insurance barriers limited access in this case. Instead, the patient responded well to 2 cycles of hyperfractionated cyclophosphamide, vincristine, adriamycin, dexamethasone, methotrexate, and cytarabine plus venetoclax, demonstrating an alternative when novel agents are unavailable.

Among 66 cases studied by Fay et al,^5^ only 19 included photographs. All showed lesions along Langer’s lines, similar to the truncal pattern in our patient (Fig 1, *C*). In 79% of these 19 cases, blood involvement was present at diagnosis, suggesting systemic disease.^4^^,^^5^ Notably, lesion color was rarely described, with only 1 figure showing purple lesions.

In 33 cases analyzed by Cota et al,^6^ skin involvement was mostly plaques and nodules on the trunk, with only 2 facial cases.^2^ Our patient’s brown patches deviate from the usual bruise-like morphology. Julia et al,^4^ in 90 BPDCN cases, also reported disseminated purple patches and nodules on the trunk.^5^ Hyperpigmented patches, as seen here, are rarely documented.

The patient’s age is also noteworthy. BPDCN typically affects children aged 6 to 12 years and adults >60 years (Cuglievan et al^3^). Fay et al reported cases from ages 20 to 90 years; Cota et al^6^ 30 to 89 years; and Julia et al 8 to 103 years, with a mean of 67.2 years. Our patient, at 18 years, falls outside typical peaks, underscoring the importance of prompt, thorough evaluation.

Given the unusual presentation, young age, and lack of systemic symptoms, this case adds to the evolving phenotype of BPDCN. Future research should further define cutaneous variants to refine diagnosis and improve early recognition.

## Conflicts of interest

None disclosed.
